# Special Issue “Recent Advances in Flame-Retardant Polymers and Composites”

**DOI:** 10.3390/molecules26206167

**Published:** 2021-10-13

**Authors:** Nam Kyeun Kim, Oisik Das

**Affiliations:** 1 Centre for Advanced Composite Materials, Department of Mechanical Engineering, The University of Auckland, Auckland 1142, New Zealand; 2 Structural and Fire Engineering Division, Department of Civil, Environmental and Natural Resources Engineering, Luleå University of Technology, 97187 Luleå, Sweden; oisik.das@ltu.se

The flame-retardant performance of materials has become an increasingly crucial factor for society across a broad range of applications in aircraft, automobiles, civil infrastructure, and consumer products. In particular, recent tragic fire incidents, such as the Grenfell Tower fire (2017) and Bushfire crisis in Australia (2020), have emphasised the necessity of providing more effective flame-resistant and structurally sound material systems to enhance fire safety in the construction and transportation industries. Hence, both national and international fire regulations for the material systems are becoming more stringent, limiting the usage of many polymeric products. In particular, polymeric composites and advanced polymers that have superior specific strength and physical properties are very susceptible to fire. Furthermore, polymers produce combustible gases, liquids, char and smoke with dripping that can be hazardous. Various flame-retardants have been developed and employed to reduce flammability, but their mechanical and physical performances have been compromised. Moreover, the release of toxic gases during the flame-retardant reaction in the gas phase is still one of the critical flammability issues. Therefore, research on the development of novel flame-retardant polymers and composites to enhance fire safety and environmental sustainability with strength is inevitable.

Scientists and researchers from different nations have contributed to this Special Issue and published a total nine papers including eight research papers and one review paper. The Special Issue covers recent research works in the fields of flame-retardant performance of polymers and composites, which can be achieved by innovative materials and processing. It also includes the development of natural resources-based flame-retardants and novel flame-retardant treatment techniques on natural fibres and biopolymers. Characterisation of the fire reaction and the mechanical properties of the flame-retarded polymers and composites are demonstrated in the published articles. Furthermore, the Special Issue highlights analytical and numerical simulations of thermal and combustion properties.

**Paper 1:** Ali, I.; Kim, N.K.; Bhattacharyya, D. Effects of Graphene Nanoplatelets on Mechanical and Fire Performance of Flax Poly-propylene Composites with Intumescent Flame Retardant. *Molecules* **2021**, *26*, 4094 [1].

The combined effects of graphene nanoplatelets and intumescent flame retardants on mechanical and fire reaction properties were investigated and a fire numerical model was developed to simulate heat release rates of flax polypropylene composites.

**Paper 2:** Schirp, A.; Dannenberg, J. Durability of Flame-Retarded, Co-Extruded Profiles Based on High-Density Polyethylene and Wheat Straw Residues. *Molecules* **2021**, *26*, 3217 [2].

Thermoplastic profiles for façade applications based on high-density polyethylene, wheat straw particles, and fire-retardants were extruded and their fire reaction properties before and after artificial weathering were evaluated.

**Paper 3:** Jiang, L.; Berto, F.; Zhang, D. Pyrolysis Kinetics and Flammability Evaluation of Rigid Polyurethane with Different Isocyanate Content. *Molecules* **2021**, *26*, 2386 [3].

Kinetic triplets, such as activation energy, pre-exponential factor and reaction order, of polyurethanes (PUs) with different isocyanate contents were obtained using Kissinger and Generic Algorithm methods. Furthermore, the effects of isocyanate contents on thermal stability of PUs were investigated by a cone calorimeter and differential scanning calorimetry.

**Paper 4:** Jafari, I.; Shakiba, M.; Khosravi, F.; Ramakrishna, S.; Abasi, E.; Teo, Y.S.; Kalaee, M.; Abdouss, M.; Ramazani, S.A.A.; Mora-di, O.; et al. Thermal Degradation Kinetics and Modeling Study of Ultra High Molecular Weight Polyethylene (UHMWP)/Graphene Nanocomposite. *Molecules* **2021**, *26*, 1597 [4].

Graphene nanosheets were incorporated into ultra-high-molecular weight polyethylene (UHMWPE) and their influences on the thermal behaviour and degradation kinetics of UHMWPE/graphene nanocomposites were investigated.

**Paper 5:** Shakiba, M.; Kakoei, A.; Jafari, I.; Rezvani Ghomi, E.; Kalaee, M.; Zarei, D.; Abdouss, M.; Shafiei-Navid, S.; Khosravi, F.; Ramakrishna, S. Kinetic Modeling and Degradation Study of Liquid Polysulfide Resin-Clay Nanocomposite. *Molecules* **2021**, *26*, 635 [5].

In this study, liquid polysulfide (LPS)/clay nanoparticles (CNPs) composites were prepared, and the presence of CNPs and their interactions with the LPS matrix were assessed via FTIR, XRD, FESEM, and EDX analyses. The TGA and DTG analyses were also used to model the thermal degradation of the neat LPS as well as the LPS/CNPs nanocomposites using Kissinger and OFW methods.

**Paper 6:** Bano, S.; Husain, F.M.; Siddique, J.A.; Alharbi, K.H.; Khan, R.A.; Alsalme, A. Role of Copper Oxide on Epoxy Coatings with New Intumescent Polymer-Based Fire Retardant. *Molecules* **2020**, *25*, 5978 [6].

The epoxy coating of intumescent flame retardant (IFR) was equipped by introducing *N*-ethanolamine triazine-piperizine, a melamine polymer (ETPMP), ammonium polyphosphate (APP), and copper oxide (CuO) in multiple composition ratios. The synergistic action of CuO on IFR coatings amplified the LOI results and enhanced the V-0 ratings in the UL-94V test.

**Paper 7:** Gebke, S.; Thümmler, K.; Sonnier, R.; Tech, S.; Wagenführ, A.; Fischer, S. Suitability and Modification of Different Renewable Materials as Feedstock for Sustainable Flame Retardants. *Molecules* **2020**, *25*, 5122 [7].

Wheat starch, wheat protein, xylan and tannin were modified with phosphate salts in molten urea. The functionalization led to the incorporation of phosphates (up to 48 wt.%) and nitrogen (up to 22 wt.%). The fire testing results indicated that these modified biopolymers can provide the same flame-retardant performances as commercial compounds currently used in the wood fibre industry.

**Paper 8:** Das, O.; Capezza, A.J.; Mårtensson, J.; Dong, Y.; Esmaeely Neisiany, R.; Pelcastre, L.; Jiang, L.; Xu, Q.; Olsson, R.T.; Hedenqvist, M.S. The Effect of Carbon Black on the Properties of Plasticised Wheat Gluten Biopolymer. *Molecules* **2020**, *25*, 2279 [8].

The research investigated the effect of carbon black as a filler in glycerol-plasticised gluten to prepare gluten/CB biocomposites and determined their mechanical, morphological, fire and chemical properties.

**Paper 9:** Ghomi, E.R.; Khosravi, F.; Mossayebi, Z.; Ardahaei, A.S.; Dehaghi, F.M.; Khorasani, M.; Neisiany, R.E.; Das, O.; Marani, A.; Mensah, R.A.; et al. The Flame Retardancy of Polyethylene Composites: From Fundamental Concepts to Nanocomposites. *Molecules* **2020**, *25*, 5157 [9].

Flame-retardant additives for polyethylene (PE) were reviewed concerning their impacts on mechanical properties. Moreover, the role of nanotechnology for more efficient flame-retardant PE systems was discussed and recommendations were given on implementing strategies that could help incorporate flame retardancy in the circular economy model.

The editors would like to express sincere appreciation to all the authors and reviewers for their contribution to this Special Issue “Recent Advances in Flame-retardant Polymers and Composites” of *Molecules*. We also thank the MDPI assistant team for their editorial supports.
